# Salivary biomarkers in cancer detection and management

**DOI:** 10.3389/abp.2025.14410

**Published:** 2025-10-20

**Authors:** Aneeq Munsif, Alicja Nowaczyk, Łukasz Fijałkowski, Saba Riaz, Amer Jamil

**Affiliations:** ^1^ Department of Biochemistry, University of Agriculture Faisalabad, Faisalabad, Pakistan; ^2^ Faculty of Pharmacy, Ludwik Rydygier Collegium Medicum in Bydgoszcz, Nicolaus Copernicus University in Toruń, Toruń, Poland

**Keywords:** biomarkers, cancer, salivary biomarker, cancer management, cancer detection

## Abstract

Studies have identified specific salivary biomarkers associated with different types of cancer, including oral, lung, and pancreatic cancers. These biomarkers can be proteins, DNA fragments, or other molecules that indicate the presence or progression of the disease. Saliva-based cancer detection offers the potential for earlier diagnosis, leading to better treatment outcomes. Additionally, salivary biomarkers can help tailor treatment plans to individual patients, improving their chances of successful recovery.

## Introduction

The uncontrolled proliferation and spreading of abnormal cells throughout the body is the foundation of cancer. More than 200 distinct forms of cancer exist, and they are all called after the organ or tissue from which they first arise. Breast cancer, prostate cancer, skin cancer, lung cancer, and colon cancer are a few prevalent forms (Adams et al., 2000; Addisu et al., 2024; Al Kawas et al., 2022; Skallevold et al., 2021).

Early diagnosis and identification can help in treating the cancer more effectively. Salivary fluid biomarkers are one of the body fluids employed in this procedure, along with physical examinations, laboratory testing, imaging procedures (such as MRIs, CT scans, and X-rays), biopsies, and other methods. The salivary glands secrete saliva, a slightly acidic oral fluid with a pH of 6–7. The saliva composition is 94%–99% water, 0.3% proteins, various cellular components, and 0.2% organic and inorganic substances (Tiwari, 2011; Kaczor-Urbanowicz et al., 2019). Saliva also contains omics molecular biomarkers, which are found in blood and urine and can be used to identify and track several cancer types, including pancreatic, gastric, lung, and oral cancer (Kaczor-Urbanowicz et al., 2019; Buzalaf et al., 2012; Vukosavljevic et al., 2014).

The main three salivary glands—the parotid, submandibular, and sublingual including the small salivary glands make up human saliva, a complex and dynamic body fluid (Yoshizawa et al., 2013). Saliva carries many important biological functions, including defense, food digestion, lubricating tissues, and clearing the mouth of food particles, and bacteria. Furthermore, saliva is the primary biological factor preventing tooth demineralization. Saliva serves as a buffer, diluting and neutralizing the acids from meals and the bacterial metabolism of the biofilm (Ahsan, 2019). The teeth’s protective layer against mineral loss, the acquired enamel pellicle, is likewise influenced by saliva (Yoshizawa et al., 2013; Buzalaf et al., 2020). Saliva’s excessively high hydroxyapatite content inhibits demineralization and encourages remineralization of dental tissues when the pH of the oral environment changes (Ahsan, 2019).

The National Institutes of Health (NIH) (Podzimek et al., 2016) defines a biomarker as a trait that can be consistently examined and assessed to serve as an indication of pathogenic processes, normal biological processes, or pharmacological responses to therapy. The identification and measurement of biomarkers found in body fluids is crucial for tracking the state of hereditary susceptibilities, pathological illnesses, and reactions to treatments and environmental factors in both clinical practice and research (Ahsan, 2019; Kaczor-Urbanowicz et al., 2017).

Saliva has been identified as a crucial body fluid for the identification of molecules that may serve as biomarkers for a range of systemic pathologies, including neurological, vascular, endocrine, cancer, and psychiatric disorders, as well as oral diseases like periodontitis and caries (Dawes and Wong, 2019; Helmerhorst and Oppenheim, 2007). In this context, it is important to emphasize that saliva and the acquired enamel pellicle are useful tools for biomarker identification. It contains around 3000 proteins, approximately half of which are similarly found in blood (Ahsan, 2019). In addition, when markers are present in both saliva and blood, they are usually found in higher concentrations in the latter, and patients prefer to donate saliva since it may be taken easily, painlessly, and without intrusive procedures. Saliva collection requires fewer steps and discomfort than urine or blood collection, which increases patient involvement—whether the patient is a child, an adult person, or someone with a disability (Gabryel-Porowska et al., 2014; Kaczor-Urbanowicz et al., 2017).

Saliva collection and processing also have low biological risk and low operating costs because they do not require workers or specialized equipment (Schulz et al., 2013). Saliva from each gland separately or may be needed, depending on the purpose of the investigation. However, for biomarker analysis, whole saliva is typically the best option. Sputum or spit, suction, and drainage are the methods most used to gather total saliva. Saliva can be collected in its entirety either while at rest (unstimulated saliva) or upon chewing, taste, or the application of pharmacological stimuli. Excessive caution should be used when collecting stimulated saliva since continuous stimulation of salivary flow may lead to insufficient glycoprotein glycosylation. Specialized tools, like the Lashley Cup, are used to do this (Ahsan, 2019). Preserving biomarkers until analysis is possible is another matter that needs to be addressed. Salivary proteins appear to degrade more quickly than serum proteins (Siqueira and Dawes, 2011; Goldoni et al., 2021). It is necessary to gather and preserve saliva to preserve the sample’s protein content. When assessing the stability of salivary biomarkers, proteolysis and incomplete protein glycosylation processes are important aspects to consider (Holmberg and Hoffman, 2014; Page et al., 2021).

Saliva contains some biomarkers, like proteins, DNA, RNA, and microRNAs, which can reveal important information about the existence and spread of cancer (Ahsan, 2019). These indicators may indicate irregular protein expression, genetic changes, or the release of chemicals particular to tumors into the saliva. Early cancer detection—even before symptoms appear—is made feasible by the analysis of salivary biomarkers (Ahsan, 2019; Sembler-Møller et al., 2020). Analyzing salivary biomarkers in-depth has enormous potential to improve cancer treatment. It makes it possible to create precise and sensitive diagnostic tools that enable early identification and action. Furthermore, salivary biomarkers can be utilized to track the course of a disease and the response to treatment (Ahsan, 2019), informing treatment choices and enhancing patient care. Particularly for those with a high risk of acquiring specific cancers, saliva collection is particularly appropriate for routine screening and monitoring due to its non-invasive nature.

The purpose of this review is to evaluate the potential mechanisms by which distant tumors influence changes in salivary biomarker profiles and cancer therapy, as well as to describe recent advancements in salivary biomarkers utilized for systemic cancer diagnosis. This will help raise awareness of the difficulties among researchers and clinicians with the hope that collaborative efforts in this field will lead to the generation of good solutions more swiftly.

## Materials and methods

Dataset: The literature query was analyzed using PRISMA (Preferred Reporting Items for Systematic Review and Meta-Analyses) guidelines (Gug et al., 2019).

Literature databases and search scheme: Searches were conducted using a combination of keywords, including salivary biomarkers, cancer, non-invasive cancer screening, therapeutic efficacy, and innovations in analytical techniques. The set of databases included Science Direct, Scopus, and PubMed. Records were identified for full-text scientific articles, published only in chemistry, medicine, toxicology or analytical journals, between 2020 and 2024. We also manually searched the bibliography of selected articles, reviews, meta-analyses, and practical tips. The 77 articles selected have been mutually agreed upon by the authors.

## Results

**FIGURE 1:**
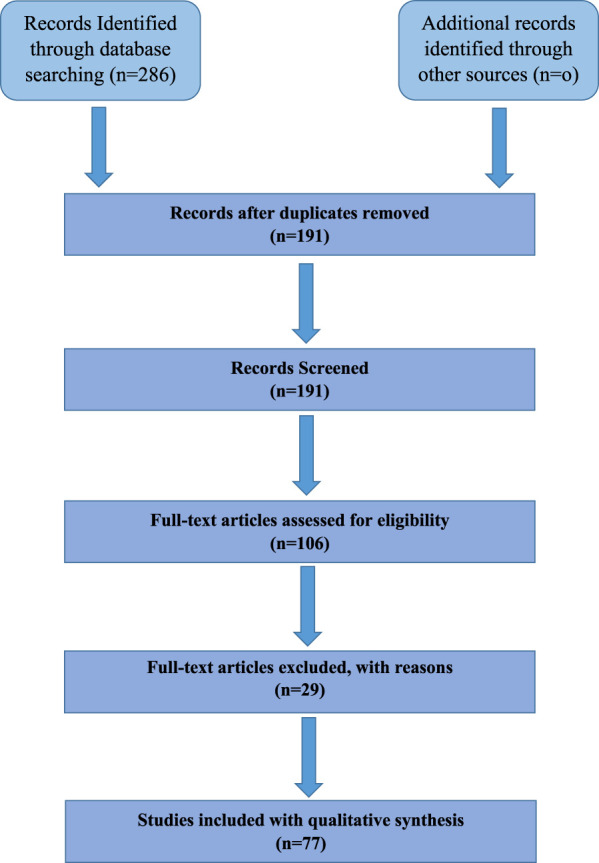
PRISMA flow diagram outlining the study selection process for this systematic review. A total of 286 records were initially identified through database searches (PubMed, Scopus, Science Direct). After removing 95 duplicates, 191 records remained for title and abstract screening. Of these, 106 full-text articles were assessed for eligibility, with 24 being unavailable and 85 excluded for irrelevance or lack of sufficient data. Finally, 77 studies were included in the qualitative synthesis.

**FIGURE 2:**
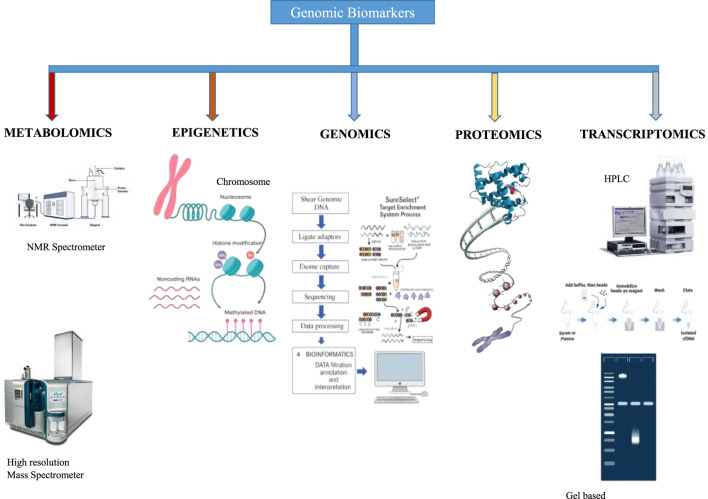
Genomic Biomarker Discovery: Advanced Methods and Technologies. This figure illustrates the powerful methods used in genomic biomarker discovery, each revealing unique insights into the complexities of life (Page et al., 2021).

The results of the systematic review are based on a comprehensive literature search conducted across PubMed, Scopus, and Science Direct. The inclusion process followed PRISMA guidelines to ensure transparency and reproducibility. The following flow diagram summarizes the study selection process, from identification to final inclusion (Figures 1, 2).

### Saliva as a monitoring and diagnostic tool

Saliva contains different biological components (enzymes, proteins, and nitrogenous products etc.) including various kinds of electrolytes like K, Na, Cl, HCO_3_, and P (Bahn et al., 2015). These biofluids may act as buffer, lubricant, antimicrobial activity, digestion, and enamel protection and removal (Bahn et al., 2015). The key information related to saliva is given in Table 1.

**TABLE 1:** Key information related to saliva.

Aspect	Key information
Function	Digestion, dental protection, antimicrobial activity, buffering effect, and lubrication
Gland Type	Major salivary glands (parotid, submandibular, sublingual) account for over 90% of saliva secretion; minor glands found in oral mucosa and palate
Secretion Mechanism	Mechanisms governing salivary gland secretion are not fully understood
Variability	Volume of saliva is not standardized; analyte levels can vary based on sampling/collection time and method
Advantages of Salivary Collection	Simple, stress-free, reproducible, and non-invasive; neither specialized physicians nor unique storage conditions are needed
Stability	Saliva is stable over time, does not clog, and allows for large sample amounts
Collection Methods	To acquire a greater sample volume, traditional procedures include scratch collection or stimulation techniques (mechanical, gustatory, or olfactory)
Flow rates	Unstimulated saliva flow rate >0.1 mL/min considered normal: stimulated saliva flow rate >0.2 mL/min
Salivaomics	Investigative approach covering genomics, transcriptomics, metabolomics, proteomics, and microbiomics
Diagnostic Tools	Saliva used in pharmacology, medicine, dentistry; “salivaomics” explores genome, RNA, metabolite profiles, proteins, and microbial population
Epigenetic Changes	DNA methylation in salivary genome associated with systemic diseases like chronic kidney disease and respiratory allergies
MicroRNA Detection	Salivary microRNA segments used for diagnosing disorders; altered miRNA profile linked to conditions such as schizophrenia, autism spectrum disorder, and Sjogren’s Syndrome
Transcriptome	Saliva contains about 117 mRNA species and 3000 mRNA transcripts, providing insights into cell processes and disease markers

Saliva is a dynamic body fluid that can have several different compositions depending on a few complex conditions. Different environmental conditions and certain personal habits can affect salivary composition (e.g., eating and drinking, smoking, sports participation) (Beaver et al., 2014). Drugs with low water solubility that cannot enter the salivary system through, the circulatory system and oral cavity-based enzymatic activity that can generate secondary metabolites are additional factors. The primary actions that may be taken to reduce confounding effects in the salivary analysis are collecting procedures and standardization of analytical techniques (Bahn et al., 2015).

### Biomarkers in saliva

Multiple types of salivary biomarkers have been identified for systemic and oral disorders: lipids, metabolites, proteins associated with risk and progression, microbes, DNA, coding, and noncoding RNA (Table 2). The development of high-throughput technologies like transcriptomics, proteomics, metabolomics, lipidomics, and microbiomics have led to a significant rise in the finding of these biomarkers. The term “salvaomics” refers to the synthesis of many “omics” methodologies for the investigation of saliva and its components (Newman et al., 2014; Ahsan, 2019).

**TABLE 2:** Overview of salivary biomarker categories for cancer detection.

Biomarker type	Examples	Detection method	Associated cancers	Notes
mRNA	IL-8, IL-1B, DUSP1	qPCR, Microarrays	Oral, Lung	High sensitivity and specificity in OSCC
miRNA	miR-31, miR-200a, miR-184	qRT-PCR	Oral, Pancreatic, Breast	Stable, found in exosomes
Protein	HER2, CD44, TNF-α	ELISA, MS, IHC	Oral, Breast, Lung	Degrade easily; protease inhibitors improve stability
Metabolite	Polyamines, Organic acids	CE-TOF-MS	Oral, Pancreatic	Reflects metabolic shifts in tumor biology
Microbiota	N. elongata, S. mitis	16S rRNA seq, qPCR	Pancreatic, Gastric	Microbial signatures linked to systemic cancers

Since preserving an accurate image of the real physiological state depends on stabilizing these molecular markers, protease and RNase inhibitors are found in extraction buffers and are utilized in many treatments. It is now becoming evident that large-scale salivary samples may conceal physiologically significant signals from distinct cell or metabolite subpopulations. To obtain more precise information from saliva in the future, ultrasensitive detection techniques or single-cell technologies would be needed (Aarthy et al., 2015).

### Transcriptome

Messenger RNA (mRNA), piwi-interacting RNA (piRNA), and micro-RNA (miRNA) are among the many RNA transcripts (Table 3) found in saliva (Aarthy et al., 2015; Russo et al., 2018; Newman et al., 2014). Studies on the salivary transcriptome mostly concentrate on mRNA and microRNA (miRNA), which are found in oral cavity cells that are separated from the original cells (Helmerhorst and Oppenheim, 2007). Noncoding RNAs (ncRNAs) are becoming more and more prominent as novel regulators of a wide range of biological processes, including oncogenesis and tumor formation. Compared to messenger RNAs (mRNAs), these molecules are less vulnerable to ribonuclease (RNase) degradation due to their small size, which makes them reasonably stable in a variety of bodily fluids (Mathew et al., 2019). Using gene microarray and quantitative real-time PCR (qRT-PCR) technologies, several mRNA and miRNA candidates with high sensitivity and specificity were identified in lung cancer (Aarthy et al., 2015), pancreatic cancer (Peres et al., 2019), and breast cancer (Aarthy et al., 2015; Adams et al., 2000; Addisu et al., 2024; Al Kawas et al., 2022; Newman et al., 2014; Skallevold et al., 2021).

**TABLE 3:** Salivary RNA biomarkers against cancer.

S. No.	RNA biomarker	Type of RNA	Used against	Remarks, if any
1.	mRNA	interleukin-8 (IL-8), interleukin-1B (IL-1B), DUSP1, OAZ1, S100P, SAT, and H3F3A	These mRNA biomarkers have exhibited varying degrees of sensitivity and specificity in detecting OSCC	These mRNAs may originate from the tumor tissue or be induced by the tumor
2.	miRNA	miR-125a, miR-200a, miR-31, miR-184, miR-27b, and miR-7	miRNAs are a significant class of non-coding RNAs that exhibit substantial fold changes in expression in OSCC.	These miRNAs can act as oncogenes or tumor suppressors
3.	circRNA	CDR1as and ci-mcm5	circRNAs like CDR1as and ci-mcm5 have been associated with tongue cancer and early oral neoplasia, respectively	Circular RNAs are a novel class of non-coding RNAs that can act as miRNA sponges and transcriptional regulators

To validate the significance of salivary miRNAs as disease markers, further preclinical and clinical research is required, despite these encouraging discoveries. Standardizing salivary exosome miRNA detection and analysis will be very important. Additionally, scientists need to come up with ways to differentiate salivary miRNA signals from tumor or salivary cells that originated in immune cells. This is significant because inflammation, whether local or systemic, can change miRNA expression and produce variability, even in the same individual (Aarthy et al., 2015). Future study data will be useful for cross-referencing with the massive catalog of extracellular noncoding RNAs found in a variety of illnesses, known as the miRNA database, or miRandola (Kassebaum et al., 2015; Patil et al., 2019).

### Proteome

The salivary proteome (Table 3), consisting of over 2000 proteins and peptides, plays a crucial role in various biological functions within the oral cavity. Proteome plasma, 20%–30% present in saliva, suggesting a possible connection between blood and salivary components (Aarthy et al., 2015). Saliva is being investigated as a stand-in for disease detection because of this close closeness. Salivary proteins can degrade quickly, which restricts their application in diagnostics. Salivary proteins can be stabilized with protease inhibitors, allowing for a two-week storage period (Aarthy et al., 2015; Almutairi et al., 2023; Azevedo et al., 2018; Hoesel and Schmid, 2013; Ma et al., 2024; Mitra et al., 2016; Srivastava et al., 2023; Xu et al., 2020; Fillies et al., 2006).

Salivary proteins are generally analyzed by mass spectrometry (MS), specifically surface-enhanced laser desorption/ionization time-of-flight (SELDI-TOF-MS) (Hegde et al., 2019; Ahsan, 2019). This method, which has been used to identify breast cancer biomarkers and distinguish between orthodontic treatment pre- and post-treatment, gives reliable profiles for healthy controls in a variety of diagnostic scenarios. When paired with mass spectrometry, two-dimensional gel electrophoresis (2DE) has demonstrated both sensitivity and specificity in identifying biomarkers for breast and lung cancer (Ishikawa et al., 2016; Adams et al., 2000; Addisu et al., 2024; Al Kawas et al., 2022; Skallevold et al., 2021) (Table 4).

**TABLE 4:** Salivary protein biomarkers against different types of cancers.

S. No.	Protein biomarker	Type of protein	Used against	Techniques used
1.	Cell surface glycoproteins	HER2, CD44, CD44sol and CA-125	Oral Squamous Cell Carcinoma (OSCC), Breast Cancer	mass spectrometry, immunohistochemistry and ELISA
2.	Cytoskeleton fragments	Cytokeratins (CK) 8, 18, 19	Oral Squamous Cell Carcinoma (OSCC)	
3.	Intracellular proteins	Mac-2 binding protein and salivary Zinc Finger Protein 510	Oral Squamous Cell Carcinoma (OSCC)	
4.	Proteases	Matrix Metalloproteinases (MMPs) like MMP-1, MMP-3, MMP-9	Oral Squamous Cell Carcinoma (OSCC), Lung Cancer	
5.	Inflammation-related proteins	NF-κB, AP-1, TNF-α, IL-6, IL-8, IL-1, COX-2, TGF-β	Oral Squamous Cell Carcinoma (OSCC), Lung Cancer	
		VEGF, EGF, and CEA	Breast Cancer	

A promising optical method for the diagnosis of cancer is Raman spectroscopy (RS), more precisely the examination of saliva protein surface-enhanced Raman spectroscopy (SERS). Research (Feng et al.) indicates that the utilization of diagnostic algorithms in conjunction with SERS can provide a non-invasive, label-free approach to the diagnosis of breast cancer (Ahsan, 2019; Adams et al., 2000; Addisu et al., 2024; Al Kawas et al., 2022).

However, the research on the utility of OSCC-related biomarkers in lung and breast cancers is still limited. More extensive validation studies are needed to determine the specificity and sensitivity of these biomarkers across different cancer types (Adams et al., 2000; Addisu et al., 2024; Al Kawas et al., 2022).

### Metabolome

The study of metabolomics involves the identification and quantification of minute metabolites produced during the biological sample metabolism, including body fluids, cells, and tissues. The global broad view of the metabolic state, called Metabolome, provides fresh insights into the pathophysiologic mechanisms underlying many illnesses. By keeping an eye on the levels of endogenous metabolites, it makes biomarker identification possible (Hsu et al., 2019). Nucleic acids, lipids, amino acids, peptides, vitamins, organic acids, thiols, and carbohydrates are examples of endogenous metabolites that might be useful in the identification of biomarkers and the monitoring of the progression of disease (Ahsan, 2019; Monea et al., 2018).

In 2010, Sugimoto et al. (2010), Wang et al. (2016) used capillary electrophoresis time-of-flight mass spectrometry (CE-TOF-MS) to identify signals specific to cancer in saliva metabolites. Using saliva samples from patients with pancreatic, breast, oral, and periodontal diseases as well as healthy controls, they conducted thorough metabolite research. AUCs (High areas under the receiver operating characteristic curves) found 57 main metabolites which can precisely predict the chance of being affected by a particular disease (Gabryel-Porowska et al., 2014). Based on MS, other salivary metabolites have been found to distinguish patients with neurodegenerative dementia and oral squamous cell carcinoma (Ahsan, 2019) from controls (Sugimoto et al., 2020).

According to different studies, salivary polyamine level was significantly higher in oral cancer. These polyamines were associated with tumor growth and spread (Mager et al., 2005). Hsu et al. established the significance of the polyamine pathway in the development of oral cancer and confirmed the rise of polyamine and its intermediate metabolites (Yang et al., 2015; Lips et al., 2017).

### Microbiota

Recent advancements in next-generation sequencing (NGS) made possible identification of approximately 19,000 phylotypes in the oral cavity (Ahsan, 2019). Additionally, data indicates that oral disorders like periodontitis (Thenisch et al., 2006), caries (Phantumvanit et al., 2018), and cancer (Ahsan, 2019) can be caused by bacteria and microbes.

The identification of bacterial taxa was formerly accomplished by culturing. But these techniques fall short of accurately describing the diversity of the oral microbiome. DNA-DNA hybridization techniques and Polymerase Chain Reaction (PCR) technology are frequently used to describe oral microbiota. But these techniques can only indicate small changes in a tissue’s microbiota (Mager et al., 2005; Mejàre et al., 2014).

Farrell et al., showed that pancreatic cancer patients may be distinguished from healthy individuals based on the presence of *N. elongata* and *S. mitis* in their saliva by using microarray and qPCR (Gao et al., 2014). By using high-throughput sequencing to sequence the microbial small subunit ribosomal RNA (16S rRNA) gene, Torres et al. (Gao et al., 2014) achieved similar results. In addition to causing stomach lining irritation, *Helicobacter pylori* (*H. pylori*) has been linked to gastric cancer. Saliva has highly sensitive markers for *H. pylori* metabolites (Ahsan, 2019).

### How do saliva biomarkers relate to distant tumors?

Prior studies have demonstrated the potential for the identification of multiple discriminatory salivary biomarkers in the event of systemic tumors, including but not limited to pancreatic cancer, breast cancer (Twetman, 2016), lung cancer (Ishikawa et al., 2016), or ovarian cancer. None of those, however, clarify how a cancer that is restricted to areas other than the mouth could affect salivary biomarker patterns. Lau et al. showed using a breast cancer cell model that salivary gland cells can interact with breast cancer exosome-like microvesicles, modifying the makeup of the exosome-like microvesicles that are formed. Researchers found that salivary gland cells secreted microscopic vesicles that resembled exosomes and contained mRNAs and proteins (Adams et al., 2000; Addisu et al., 2024; Al Kawas et al., 2022; Skallevold et al., 2021).

Exosomes are small vesicles that range in width from 30 to 120 nm and are composed of proteins, lipids, mRNA, miRNA, DNA, and other materials (Ahsan, 2019; Hassanein et al., 2012). It is believed that they deliver these substances from far-off locations to every part of the body. Practically every type of cell and physiological fluid, including saliva, contains exosomes (Xiao et al., 2012). Studies have revealed that exosomes are involved in the processing and degradation of RNA (Ahsan, 2019), the spread of pathogens (Zhang et al., 2012), the promotion of tumors (Roi et al., 2019; Kaufman and Lamster, 2002), and immune function (Ahsan, 2019).

### Saliva biomarkers for cancer detection

#### Lung cancer

Lung cancer is the primary reason of death related to cancer in the US, contributing to 27% and 28% deaths in women and men respectively. According to the American Cancer Society (ACS), the United States is expected to have 234,580 new cases of lung cancer in 2024, with 116,310 in men and 118,270 in women. The ACS also estimates that 125,070 people will die from lung cancer in 2024, with 65,790 men and 59,280 women (Siegel et al., 2024). The 5-year survival rate of lung cancers is significantly lower (17%) as compared to prostate, breast, and colon carcinomas which are 99%, 89%, and 65% respectively (Ahsan, 2019; Podzimek et al., 2016; Skallevold et al., 2021).

Early diagnosis of lung cancer is crucial because it is the major leading reason of men and women death. Due to their high false-negative rate, conventional diagnosis techniques are not suitable for screening. Despite its high false-positive rate, CT is routinely utilized for early screening of lung cancer. Without the use of CT, salivary biomarkers may aid in early detection (Kaczor-Urbanowicz et al., 2017; Parisotto et al., 2010). In lung cancer diagnosis 16 putative biomarkers have been identified through research as effective means (Xiao et al., 2016), three of which—haptoglobin, calprotectin, and zinc-a-2-glycoprotein—have been shown to have high sensitivity and great specificity. The transcriptome biomarker profile, which includes the leucine zipper putative tumor suppressor 1, fibroblast growth factor receptor substrate 2, cyclin I, the EGF receptor, FGF-19, B-Raf gene, and growth regulation by estrogen in breast cancer 1, has been identified. A panel of five of these biomarkers was able to achieve a specificity of 82.81% and a sensitivity of 93.75% when it came to the diagnosis of lung cancer (Ruhl, 2012; Elashoff et al., 2012; Adams et al., 2000; Addisu et al., 2024; Al Kawas et al., 2022; Newman et al., 2014; Skallevold et al., 2021).

#### Breast cancers

The second leading cause of cancer-related deaths in the US is breast cancer, which is also the most frequent cancer type among women. In the United States, approximately 40,290 people lost their lives to this illness in 2015 (Ahsan, 2019), despite improvements in therapy. Breast cancers are often found late in life, which raises the death rate. Though the sensitivity varies according to the type of mammography, traditional screening mammography remains the gold standard for diagnosing breast cancer (Nahar et al., 2020). According to recent studies salivary biomarkers could be used with the purpose of early diagnosis and to monitor advanced disease. (Gug et al., 2019; Adams et al., 2000; Addisu et al., 2024; Al Kawas et al., 2022).

#### Pancreatic cancer

In the US, pancreatic cancer claims the lives of 44,030 people annually, accounting for 37,660 deaths (Brinkmann et al., 2011; Liang et al., 2022). Numerous biomarkers were created for pancreatic cancer (Sugimoto et al., 2010; Ahsan, 2019), but more precisely, miR-3679–5p and miR–3679–5p together can be used for the identification of pancreatic malignancies (Twetman, 2016). They can identify variations in salivary endogenous metabolite concentrations. The presence of N. elongata and S. mitis in saliva can differentiate between pancreatic cancer patients and healthy individuals with help of qPCR and HOMIM (Human Oral Microbe Identification Microarray) having specificity of 82.1% and sensitivity of 96.4% (Ahsan, 2019).

#### Gastric cancer

The second leading cause of cancer-related mortality worldwide and the fourth most common cancer overall is gastric cancer (Liang et al., 2022) More than 880,000 people lose their lives to this illness each year. Since early stomach tumors frequently show no symptoms at all or merely produce nonspecific ones, delayed detection is the main reason for the high death rate (Liang et al., 2022). Gastric cancer has been linked to deletion in malignant brain tumors 1 protein (DMBT1), triosephosphate isomerase (TPI1), and cyclostatin B (CSTB) (Liang et al., 2022; Ai et al., 2010) Many target molecules, including extracellular RNA, amino acids, proteins, and glycoproteins have been the subject of studies on salivary biomarkers (Ahsan, 2019).

## Discussion

Salivary biomarkers have emerged as a promising tool in the field of cancer management, offering a non-invasive and accessible approach to tackling this complex disease. These biomarkers, which can be derived from various molecules present in saliva, including DNA, RNA, proteins, and metabolites, have the potential to revolutionize how we detect, diagnose, monitor, and even predict the prognosis of various types of cancer.

One of the most exciting applications of salivary biomarkers is in the sphere of early cancer detection. Numerous studies have demonstrated the ability of these biomarkers to identify the presence of cancer even before clinical symptoms become apparent (Pereira et al., 2020; Bel’skaya and Dyachenko, 2024). This early detection capability is crucial, as it can lead to timely intervention and significantly improve treatment outcomes for patients. Moreover, salivary biomarkers have shown promise in differentiating between cancerous and benign lesions, aiding in accurate diagnosis, and guiding appropriate treatment strategies (Chakraborty et al., 2019; Sancesario and Bernardini, 2019).

Beyond diagnosis, salivary biomarkers have also proved valuable in monitoring the effectiveness of cancer treatments. By tracking changes in the levels of specific biomarkers during and after treatment, clinicians can gain valuable insights into the patient’s response to therapy and make informed decisions about adjusting the course of treatment as needed (Hartmann and Ledur Kist, 2018).

Perhaps most intriguing is the potential of salivary biomarkers to predict disease prognosis and the likelihood of cancer recurrence. Certain biomarker profiles have been associated with more aggressive disease trajectories or higher risks of relapse, allowing for the development of personalized treatment plans that optimize patient outcomes (Spielmann and Wong, 2011).

As the field of salivary biomarker research continues to evolve, we can expect to see even more advancements in the clinical application of these non-invasive tools. With their ability to provide valuable insights at various stages of cancer management, salivary biomarkers hold the promise of transforming the way we approach this complex and challenging disease (Langie et al., 2016).

## Conclusion

The detection and diagnosis of cancer remains challenging, especially in its early stages when symptoms are often nonspecific. The development of easy, precise, and non-invasive cancer screening, diagnosis, and management techniques requires urgent attention. Because biofluid-based detection uses molecular biomarkers and is non-invasive, it has the potential to identify diseases early in high-risk patients.

Recent developments in high-throughput omics analysis have played a major role in the identification of biofluid-based cancer-associated biomarkers. Nevertheless, despite great efforts, only a small number of biomarkers have been included into standard clinical practice, and even fewer have received approval for diagnosis or screening of the entire population.

Salivary biomarkers are becoming more widely acknowledged for their ability to track the development and response to therapy of cancer in addition to detecting it. For example, tumour regression or resistance in oral squamous cell carcinoma has been associated with dynamic variations in IL-8 or TNF-α levels throughout therapy. Similar to this, changes in miRNA profiles, including those of miR-31 and miR-200a, before and after chemotherapy can show how well a treatment is working for lung and breast cancer. It has been suggested that monitoring polyamine levels and particular exosomal RNA fragments can be used to detect minimal residual disease in gastric and pancreatic malignancies. These long-term biomarker evaluations offer a non-invasive and economical method of managing cancer in a personalized manner, allowing for prompt therapy modifications and enhancing prognosis. Challenges such as the heterogeneity of cancer pathology and technical issues with omics technologies persist, underscoring the need for further research and development (Adams et al., 2000; Addisu et al., 2024; Al Kawas et al., 2022).

The research of salivary biomarkers could undergo an enormous shift as a result of developments in nanotechnology and artificial intelligence. Point-of-care diagnostic devices that assess multi-analyte saliva profiles for rapid chairside diagnosis are being developed with machine learning algorithms. The miniaturization of lab-on-a-chip technologies, which process and analyze minute salivary samples in real-time, has made distributed cancer screening viable even in low-resource contexts. Multinational consortiums, such as the Salivaomics Knowledge Base, are also attempting to standardize the processes involved in the identification and validation of biomarkers. As these technologies develop, salivary diagnostics might soon be included into routine cancer operations, enabling earlier detection, more accurate monitoring, and better patient outcomes.
